# Using *Heading date 1* preponderant alleles from *indica* cultivars to breed high‐yield, high‐quality *japonica* rice varieties for cultivation in south China

**DOI:** 10.1111/pbi.13177

**Published:** 2019-06-17

**Authors:** Yujia Leng, Yihong Gao, Long Chen, Yaolong Yang, Lichao Huang, Liping Dai, Deyong Ren, Qiankun Xu, Ya Zhang, Kimberly Ponce, Jiang Hu, Lan Shen, Guangheng Zhang, Guang Chen, Guojun Dong, Zhenyu Gao, Longbiao Guo, Guoyou Ye, Qian Qian, Li Zhu, Dali Zeng

**Affiliations:** ^1^ State Key Laboratory for Rice Biology China National Rice Research Institute Hangzhou China; ^2^ CAAS‐IRRI Joint Laboratory for Genomics‐assisted Germplasm Enhancement, Agricultural Genomics Institute in Shenzhen Chinese Academy of Agricultural Sciences Shenzhen China

**Keywords:** major Chinese cultivated rice varieties, *Hd1* preponderant allele, association analysis, heading date, yield‐ and quality‐related traits improvement

## Abstract

*Heading date 1* (*Hd1*) is an important gene for the regulation of flowering in rice, but its variation in major cultivated rice varieties, and the effect of this variation on yield and quality, remains unknown. In this study, we selected 123 major rice varieties cultivated in China from 1936 to 2009 to analyse the relationship between the *Hd1* alleles and yield‐related traits. Among these varieties, 19 haplotypes were detected in *Hd1*, including two major haplotypes (H8 and H13) in the *japonica* group and three major haplotypes (H14, H15 and H16) in the *indica* group. Analysis of allele frequencies showed that the secondary branch number was the major aimed for Chinese *indica* breeding. In the five major haplotypes, SNP_316_(C‐T) was the only difference between the two major *japonica* haplotypes, and SNP_495_(C‐G) and SNP_614_(G‐A) are the major SNPs in the three *indica* haplotypes. Association analysis showed that H16 is the most preponderant allele in modern cultivated Chinese *indica* varieties. Backcrossing this allele into the *japonica* variety Chunjiang06 improved yield without decreasing grain quality. Therefore, our analysis offers a new strategy for utilizing these preponderant alleles to improve yield and quality of *japonica* varieties for cultivation in the southern areas of China.

## Introduction

Rice (*Oryza sativa* L.) is an important cereal crop and provides the staple food for more than half of the world's population (Tian *et al*., 2009). Achieving increases in grain yield in rice is a major focus in agriculture (Ren *et al*., 2018; Yuan, 2014). Heading date (flowering time) is one of the most important agronomic traits for regional adaptation and grain yield, and is affected by genetic and environmental factors (Li *et al*., 2015). Selection for the optimal flowering time for a particular region will make maximum use of temperature and sunlight conditions to improve yield potential (Izawa, 2007; Zheng *et al*., 2016). Therefore, detailed knowledge of the genetic factors for heading date will increase our understanding of the adaptive mechanisms of rice varieties and enable breeders to design appropriate genotypes for specific environments (Putterill *et al*., 2004; Zheng *et al*., 2016).

Rice is a short‐day (SD) flowering plant; its heading is promoted under short photoperiod conditions. In recent years, the molecular genetic pathway for SD photoperiodic regulation in cultivated rice has been well characterized. OsGI (a homolog of *Arabidopsis thaliana* GIGANTEA) is responsible for perceiving light signals and circadian clocks, and regulates the expression of *Heading date 1* (*Hd1*, an ortholog of *CONSTANS* in Arabidopsis) and *OsMADS51* (Hayama *et al*., 2002, 2003; Kim *et al*., 2007; Takahashi *et al*., 2009). *Hd1*, which encodes zinc‐finger‐type transcriptional activators with CCT domains, promotes flowering under SD conditions and represses flowering under long‐day (LD) conditions by regulating the expression of *Heading date 3a* (*Hd3a*, an ortholog of *FLOWERING LOCUS T* in Arabidopsis; Kojima *et al*., 2002; Yano *et al*., 2000). *OsMADS51*, which encodes a type I MADS‐box protein and functions upstream of *Early heading date 1* (*Ehd1*), promotes flowering under SD conditions (Kim *et al*., 2007). *Ehd1* is a B‐type response regulator, which promotes flowering by regulating the expression of *Hd3a* and independently from *Hd1* pathway (Doi *et al*., 2004). *RICE FLOWERING LOCUS T1* (*RFT1*) functions as a floral activator, belongs to the rice *FT*‐like gene family and is the closest homolog of *Hd3a* (Komiya *et al*., 2008). RFT1 and Hd3a are both mobile flowering signals but RFT1 functions under LD conditions, whereas Hd3a functions under SD conditions (Komiya *et al*., 2008). Recent research demonstrated that *RFT1* and *Hd3a* functionally diverged to control flowering time under LD and SD conditions, partly via a fine‐tuned epigenetic mechanism (Li *et al*., 2015; Sun *et al*., 2012).

Natural genetic variation provides an opportunity to identify key alleles associated with traits that can be selected to improve agronomic characteristics of crops (Lu *et al*., 2013; Zhu *et al*., 2008). For example, the pleiotropic gene *Ghd7*, which affects flowering time, plant height and spikelet number per panicle, was shown to contain an important single nucleotide polymorphism (SNP) that affects these three related traits in rice (Lu *et al*., 2012). Two SNPs in maize (*Zea mays*) *Dwarf8* were shown to be independently associated with flowering time and plant height (Thornsberry *et al*., 2001). As an important heading date gene, *Hd1* alleles have been widely used in traditional rice breeding and are also good targets for molecular marker‐assisted selection (MAS) breeding.

China has a long history of rice cultivation and a broad range of rice cultivation regions (from 18°N to 53°N), which contribute to a diversity of flowering time variants. However, the utilization of preponderant alleles of *Hd1* in modern cultivated Chinese varieties is still unclear. In this study, we selected 123 major rice varieties cultivated in China from 1936 to 2009 in order to: (i) evaluate their genetic diversity and population structure; (ii) identify the key *Hd1* alleles affecting yield‐related traits; and (iii) utilize the preponderant alleles of *Hd1* in rice breeding to improve yield and quality. The results of this study will provide valuable data and a new strategy for generating varieties more suited to specific regions, potentially improving yield.

## Results

### Wide variation of yield‐related traits was noted in the 123 major rice varieties cultivated in China

**Figure 1:**
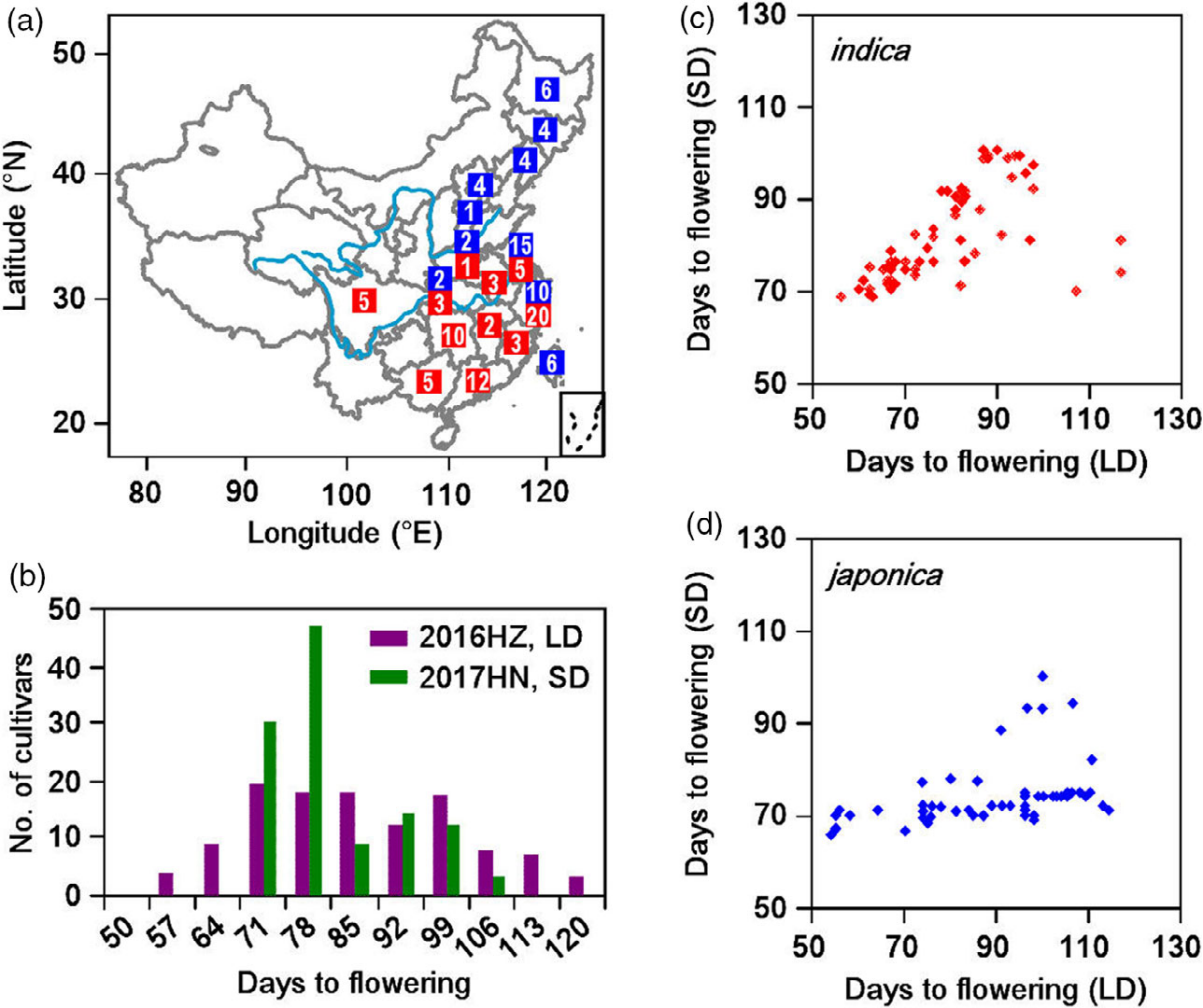
Variations in heading date and seven yield‐related traits among 123 major rice varieties cultivated in China in two conditions. (a) Geographical distribution of 123 major rice varieties cultivated in China. Blue indicates *japonica*, and red indicates *indica*. The number within each bar indicates the number of cultivars at this region. (b) Phenotypic variation of heading date in 2016 Hangzhou (long day, violet) and 2017 Hainan (short day, green). (c) Correlation analysis of heading date in *indica* varieties. (d) Correlation analysis of heading date in *japonica* varieties.

We selected 123 major rice varieties cultivated in China from 1936 to 2009, based on these varieties were the main cultivars at that time. These 123 varieties, comprising 54 *japonica* and 69 *indica* varieties, were selected to analyse the relationship between the *Hd1* alleles and eight yield‐related traits (Figure 1a, Table S1). A wide variation in heading date was observed among the 123 varieties, ranging from 54 to 117 days in Hangzhou (a LD site) and 66 to 100 days in Hainan (a SD site; Figure 1b).

The photoperiod analysis showed that most of the *indica* varieties were photoperiod insensitive, while most *japonica* varieties were not (Figure 1c, d). The other seven yield‐related traits were highly diverse among the collection. For example, tiller number (TN) ranged from 5.6 to 14.2 in Hangzhou and 5.5 to 12.0 in Hainan. Grain number per plant (GNPP) ranged from 59.5 to 247.3 in Hangzhou and 71.4 to 207.2 in Hainan. Thousand grain weight (TGW) ranged from 17.6 to 28.8 in Hangzhou and 19.6 to 33.4 in Hainan (Table 1).

**Table 1:** Descriptive statistics of eight yield‐related traits of 123 major rice varieties cultivated in China in 2016 (Hangzhou, LD) and 2017 (Hainan, SD)

Trait	Year	*Japonica* (*n* = 54)		*Indica* (*n* = 69)	
		Range	Mean ± SD	Range	Mean ± SD
HD	2016 LD	54.0–114.0	87.8 ± 17.51	56.0–117.0	78.1 ± 13.34
	2017 SD	66.0–100.0	73.9 ± 7.12	68.0–100.0	81.3 ± 9.88
TN	2016 LD	5.6–14.2	9.2 ± 1.76	5.4–16.3	9.7 ± 2.11
	2017 SD	5.5–12.0	8.2 ± 1.51	4.5–10.8	6.7 ± 1.55
PBN	2016 LD	6.3–16.3	11.4 ± 2.22	6.0–16.5	11.3 ± 2.18
	2017 SD	6.8–12.0	8.8 ± 1.21	5.2–15.8	10.3 ± 1.92
SBN	2016 LD	9.0–51.0	24.8 ± 8.58	14.3–72.5	35.8 ± 12.10
	2017 SD	10.4–41.2	21.4 ± 6.00	13.6–62.0	32.6 ± 11.00
GNPP	2016 LD	59.5–247.3	138.9 ± 37.60	84.0–370.8	186.7 ± 54.57
	2017 SD	71.4–207.2	116.9 ± 27.18	80.0–312.6	176.4 ± 53.85
TGW	2016 LD	17.6–28.8	23.9 ± 2.76	13.5–31.4	23.8 ± 3.36
	2017 SD	19.6–33.4	27.0 ± 2.37	15.0–34.9	27.1 ± 3.74
GWPP	2016 LD	15.0–46.8	29.5 ± 6.50	22.0–68.5	40.6 ± 9.28
	2017 SD	16.5–30.9	25.0 ± 3.14	15.0–43.1	30.2 ± 5.73
GWSP	2016 LD	1.5–5.4	3.3 ± 0.87	2.1–7.1	4.4 ± 1.24
	2017 SD	1.9–5.2	3.1 ± 0.59	2.3–7.8	4.7 ± 1.42

GNPP, grain number per plant; GWPP, grain weight per plant; GWSP, grain weight per single panicle; HD, heading date; LD, long day; *n*, number of cultivars tested; PBN, primary branch number; SBN, secondary branch number; SD, short day; SD, standard deviation; TGW, 1000‐grain weight; TN, tiller number.

### SSR diversity and population structure

Twenty‐eight polymorphic SSR markers, randomly distributed on the 12 rice chromosomes, were selected to evaluate the genetic diversity of the 123 cultivated varieties. A total of 128 alleles were amplified and individual SSR marker contained between 2 and 9 alleles with an average of 4.5714 alleles for each marker. The average gene diversity was 0.5835, ranging from 0.4489 to 0.7980. The average polymorphism information content (PIC) value was 0.5027, ranging from 0.3586 to 0.7735 (Table S2).

The population structure of the 123 cultivated varieties based on the SSR genotype showed that the highest log‐likelihood scores of the population structure were observed when the number of populations was set at two (*K* = 2), suggesting that these varieties can be classified into two subpopulations (Figure S1a). A neighbour‐joining tree of 123 cultivated varieties was constructed based on Nei's genetic distance, which indicated that the rice accessions could be well differentiated into *japonica* and *indica* groups (Figure S1a).

### *Hd1* exhibited a high degree of nucleotide polymorphism and protein diversity

**Figure 2:**
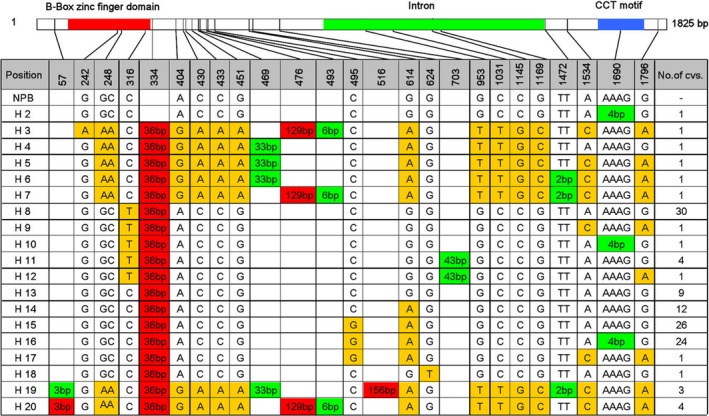
A high degree of polymorphism in the *Hd1* coding sequence in 123 major rice varieties cultivated in China. *Hd1* contains two exons (indicated in white rectangles) and two domains (red rectangle indicates the B‐Box zinc finger domain, and blue rectangle indicates the CCT motif). The *Hd1* nucleotide sequences of the 123 varieties were compared with that of cv. Nipponbare. Polymorphic nucleotides are indicated by different colours. Base substitution, deletion and insertion sites are indicated by yellow, green and red background, respectively. The number of cultivars with each type of sequence (haplotypes 2–20) is shown in the column at the right.

We analysed the *Hd1* coding region (1825 bp, which includes one intron and two exons) in the 123 cultivated varieties. The *Hd1* sequence exhibited a high degree of nucleotide polymorphism; Thirteen SNPs and 10 insertions/deletions (InDels) were detected in the coding region of *Hd1*, of which 3 SNPs were found in the zinc finger domain and 1 InDel was found in the CCT domains (Figure 2). The *Hd1* sequence of Nipponbare (NPB, haplotype 1, H1) was defined as the reference sequence. A total of 19 haplotypes, named H2 to H20, were identified in the 123 cultivated varieties (Figure 2). Haplotypes H4, H5, H8‐13, H17 and H20 belong to the *japonica* subpopulation, while haplotypes H2, H3, H6, H7, H14‐16, H18 and H19 belong to the *indica* subpopulation. The most prevalent haplotypes were H8, H13, H14, H15 and H16, which included 30, 9, 12, 26 and 24 varieties, respectively. Of the remaining haplotypes, each was found in four or fewer varieties (Figure 2, Table S1). When comparing the mean values of the eight target traits in plants with the five major *Hd1* haplotypes, significant differences were detected among the five haplotypes except for TN in Hangzhou and TGW in both locations (Table 2).

**Table 2:** Comparison of means of eight traits among the five major haplotypes

Trait	Year	H 8 (*n* = 30)	H 13 (*n* = 9)	H 14 (*n* = 12)	H 15 (*n* = 26)	H 16 (*n* = 24)	*F*	*P*
		Means ± SD	Means ± SD	Means ± SD	Means ± SD	Means ± SD		
HD	2016 LD	91.8 ± 13.34b	102.3 ± 9.08a	70.0 ± 6.38c	74.5 ± 16.93c	85.5 ± 8.41b	3.53	<0.0001
	2017 SD	75.3 ± 8.09b	72.3 ± 1.66b	74.6 ± 1.68b	75.0 ± 5.89b	91.3 ± 7.22a	5.71	<0.0001
TN	2016 LD	8.9 ± 1.24b	10.11 ± 1.71ab	9.0 ± 1.18b	10.5 ± 2.26a	9.0 ± 1.83b	1.48	0.0891
	2017 SD	8.0 ± 1.39b	9.5 ± 1.24a	6.5 ± 1.09 cd	7.3 ± 1.63bc	6.1 ± 1.45d	2.47	0.0009
PBN	2016 LD	12.1 ± 1.60a	11.2 ± 1.29ab	10.1 ± 1.50b	10.7 ± 2.36b	12.4 ± 1.61a	1.71	0.0323
	2017 SD	8.8 ± 1.21 cd	8.0 ± 0.41d	9.2 ± 1.03bc	9.9 ± 2.04b	11.5 ± 1.14a	2.68	0.0003
SBN	2016 LD	26.9 ± 7.67 cd	21.6 ± 4.42d	36.3 ± 9.32ab	30.2 ± 11.44bc	42.7 ± 11.66a	2.07	0.0059
	2017 SD	22.0 ± 5.96c	17.0 ± 3.98c	32.5 ± 6.62ab	29.5 ± 11.67b	38.3 ± 10.54a	2.95	<0.0001
GNPP	2016 LD	149.2 ± 31.57bc	128.5 ± 17.20c	178.2 ± 39.31b	161.2 ± 50.87b	220.5 ± 52.43a	2.11	0.0048
	2017 SD	119.1 ± 27.29c	96.6 ± 16.36c	164.5 ± 32.03b	163.5 ± 60.68b	206.4 ± 46.19a	3.33	<0.0001
TGW	2016 LD	23.4 ± 3.00ab	25.1 ± 2.63a	25.6 ± 2.64a	24.5 ± 1.51ab	22.6 ± 4.64b	0.92	0.5939
	2017 SD	26.7 ± 2.61bc	29.2 ± 1.75a	29.0 ± 2.45ab	27.5 ± 2.31ab	26.2 ± 5.21c	1.37	0.1393
GWPP	2016 LD	30.5 ± 5.32b	32.1 ± 4.67b	40.0 ± 6.90a	39.6 ± 9.53a	43.1 ± 9.79a	1.90	0.0135
	2017 SD	24.5 ± 2.75b	26.4 ± 2.75b	30.6 ± 5.21a	30.5 ± 5.84a	31.3 ± 5.12a	1.98	0.0089
GWSP	2016 LD	3.5 ± 0.75c	3.2 ± 0.55c	4.6 ± 1.06ab	3.9 ± 1.21bc	4.9 ± 1.19a	1.86	0.0160
	2017 SD	3.1 ± 0.55c	2.8 ± 0.40c	4.8 ± 1.02ab	4.4 ± 1.46b	5.3 ± 1.41a	3.54	<0.0001

H, haplotype; LD, long day; *N*, number of cultivars tested; SD, short day; SD, standard deviation.

Letters are ranked by Duncan test at *P *<* *0.05. The same letter within the same column represents no significant difference. *F* ratio and probability based on one‐way analysis of variance.

**Figure 3:**
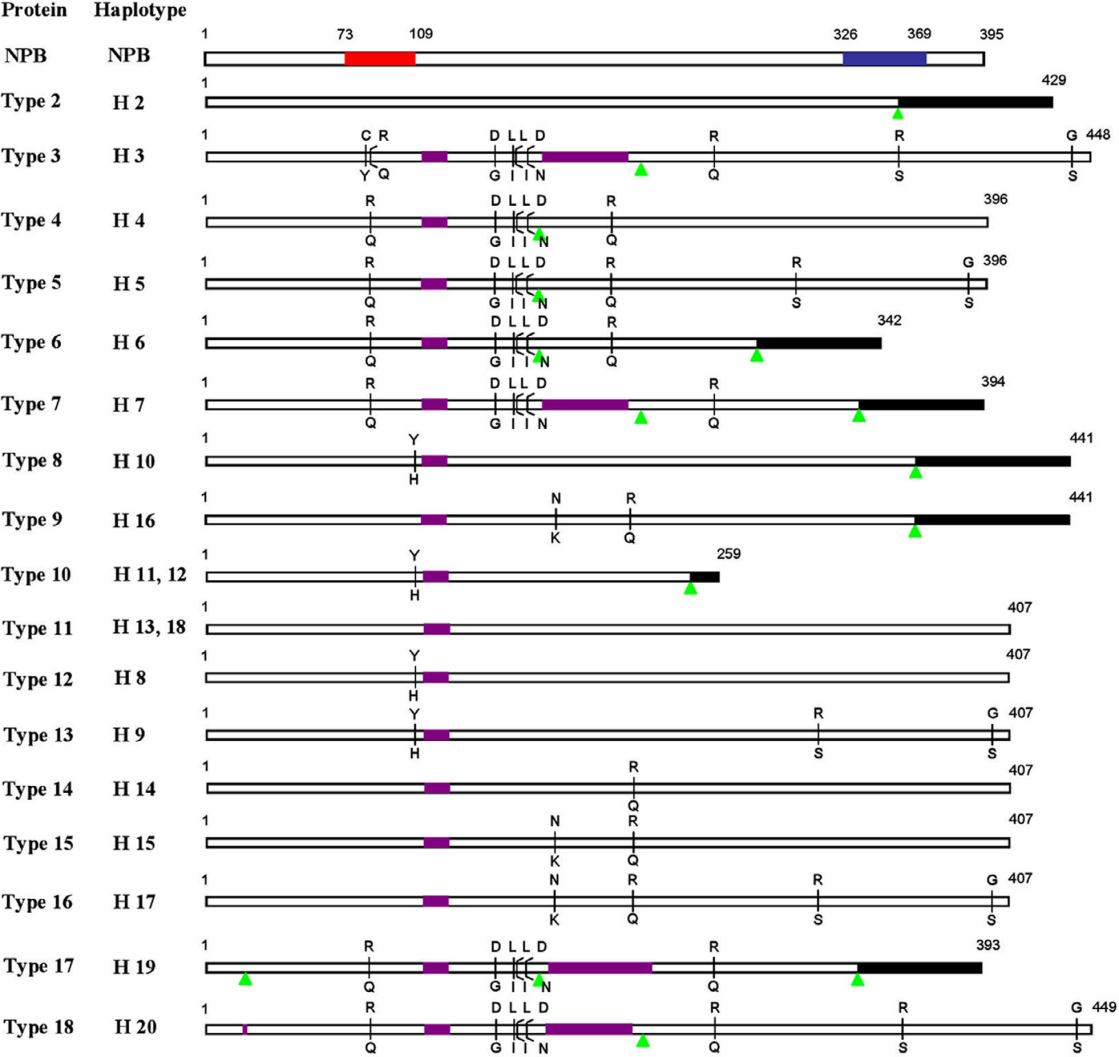
Hd1 protein diversity in 123 major rice varieties cultivated in China. The Hd1 protein sequences of the 123 major rice varieties cultivated in China were compared with that of NPB. The B‐Box zinc finger domain and CCT motif are indicated by red and blue rectangles, respectively. Purple rectangles and green triangles indicate amino acid insertion and deletion sites, respectively. Black rectangles indicate altered amino acid sequences caused by frame shifts. The letter above the vertical lines indicates NPB amino acids, and the letter below indicates the amino acid substitution.

Hd1 proteins also displayed high diversity in the present study (Figure 3). A total of 17 distinct proteins were identified, which include 10 functional proteins (types 3–5, 11–16 and 18) and 7 nonfunctional proteins (types 2, 6, 7, 8–10 and 17). Haplotypes H11 and H12, and H13 and H18 shared the same protein type. Among the 17 distinct proteins, five major protein types (types 9, 11, 12, 14 and 15) were found, types 11 and 12 belong to the *japonica* group and types 9, 14 and 15 belong to the *indica* group (Figure 3).

### The *Hd1* ^*ind*^ haplotypes displayed signatures of artificial selection

**Figure 4:**
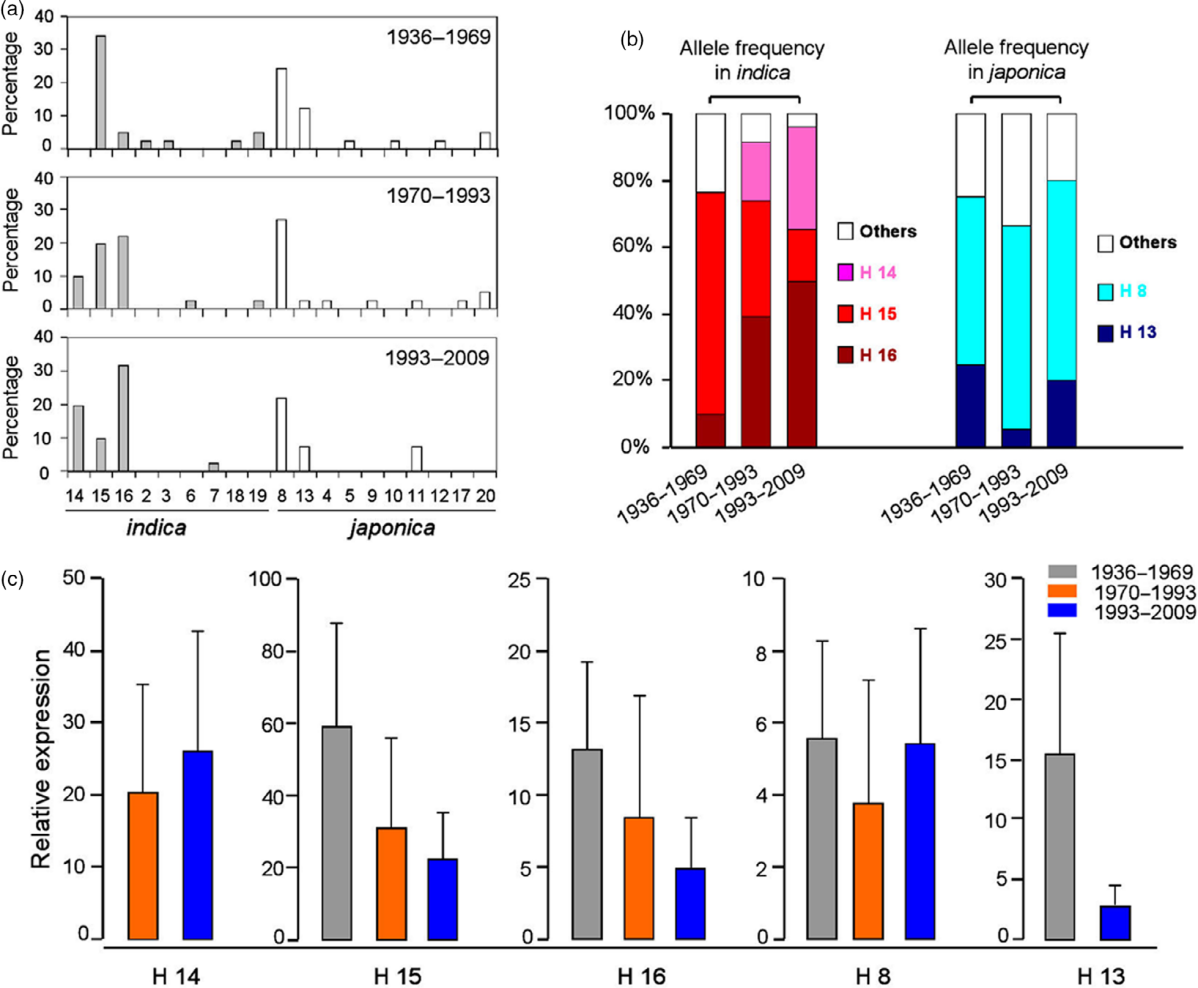
Frequency and expression of *Hd1* haplotypes in 123 major rice varieties cultivated in China. (a) Allele frequencies of 19 *Hd1* haplotypes among the three time period groups. Darker grey rectangles indicate *indica* haplotypes, and white rectangles indicate *japonica* haplotypes. (b) Allele frequencies of five major haplotypes among the three stages. Pink, red, brown, light blue, deep blue and white indicate haplotypes 14, 15, 16, 8, 13, and all other haplotypes, respectively. (c) The expression of five major haplotypes among the three stages.

In order to identify which *Hd1* haplotypes were the major types used in different years, we divided the 123 varieties into three groups according to the period of time of their release: years 1936–1969, 1970–1993 and 1993–2009 (Figure 4). Among the five major haplotypes, the *Hd1* ^*jap*^ haplotype H8 maintained a high selection frequency in all 3 groups, with allele frequencies of 50%, 61% and 60%, respectively (Figure 4a, b). Moreover, the expression level of H8 changed very little among three groups (Figure 4c). For *Hd1* ^*ind*^ haplotypes, distinct differences were noted among the three groups. For example, H15 was the major haplotype from 1936 to 1969, but H14 and H16 were the major haplotypes from 1993 to 2009 (Figure 4a). The allele frequencies of H14, H15 and H16 were 0%, 67% and 10% in the 1936–1969 group, and 31%, 15% and 50% in the 1993–2009 group, respectively (Figure 4b). Interestingly, the expression of *Hd1* ^*ind*^ alleles decreased in H15 and H16 (Figure 4c). These results indicated that the preponderant *Hd1* allele has changed in recent decades in Chinese *indica* varieties.

We further compared these 3 *indica* haplotypes to the mean values of the eight target traits; significant differences were detected in secondary branch number (SBN), indicating that the secondary branch number was the major aim for breeding of Chinese *indica* cultivars (Table 3).

**Table 3:** Comparison of eight yield‐related traits among three major *Hd1* ^*ind*^ haplotypes in varieties cultivated in 1936–1969 and 1993–2009

Trait	Year	1936–1969	1993–2009		*F*	*P*
		H 15 (*n* = 14)	H 14 (*n* = 8)	H 16 (*n* = 13)		
HD	2016 LD	78.5 ± 20.84ab	69.4 ± 6.00b	86.9 ± 9.01a	1.35	0.2660
	2017 SD	74.3 ± 5.21b	74.6 ± 1.41b	90.9 ± 6.69a	7.63	<0.0001
TN	2016 LD	11.3 ± 2.13a	8.7 ± 1.00b	8.8 ± 1.96b	1.37	0.2548
	2017 SD	7.9 ± 1.32a	6.7 ± 1.30ab	5.8 ± 1.58b	1.81	0.1104
PBN	2016 LD	10.6 ± 2.a	10.4 ± 1.47a	12.1 ± 1.30a	1.02	0.4781
	2017 SD	9.4 ± 2.03b	9.2 ± 1.22b	11.3 ± 0.93a	1.23	0.3301
SBN	2016 LD	23.7 ± 5.93b	37.9 ± 9.84a	45.1 ± 13.38a	2.62	0.0248
	2017 SD	24.3 ± 7.35c	33.1 ± 7.67b	42.0 ± 11.68a	2.46	0.0332
GNPP	2016 LD	134.9 ± 28.59b	185.4 ± 40.99a	223.9 ± 60.18a	1.95	0.0857
	2017 SD	137.2 ± 41.11b	164.1 ± 38.19b	217.78 ± 52.65a	1.90	0.0932
TGW	2016 LD	24.7 ± 1.50a	24.7 ± 1.74a	23.2 ± 5.38a	1.00	0.4902
	2017 SD	28.0 ± 1.31a	27.9 ± 1.61a	26.5 ± 6.11a	1.07	0.4353
GWPP	2016 LD	36.7 ± 6.28a	39.2 ± 7.41a	43.2 ± 9.28a	1.00	0.4934
	2017 SD	29.3 ± 6.44a	29.8 ± 6.18a	31.5 ± 5.62a	1.46	0.2177
GWSP	2016 LD	3.3 ± 0.68b	4.6 ± 1.08a	5.0 ± 1.12a	1.58	0.1717
	2017 SD	3.8 ± 1.09b	4.6 ± 1.16b	5.7 ± 1.53a	2.38	0.0384

H, haplotype; LD, long day; *N*, number of cultivars tested; SD, short day; SD, standard deviation. Letters are ranked by Duncan test at *P *<* *0.05. The same letter within the same column represents no significant difference. *F* ratio and probability based on one‐way analysis of variance.

### Haplotype 16 was the preponderant allele in Chinese *indica* breeding

An association analysis between *Hd1* haplotypes and eight yield‐related traits was conducted to identify SNP‐trait associations separately using a general linear model (GLM), which accounted for population structure data (Table S1). Five major haplotypes, H8, H13, H14, H15 and H16, were analysed. Other haplotypes were excluded because of a limited number of varieties (four or fewer). Three SNPs (SNP_316_(C‐T), SNP_495_(C‐G) and SNP_614_(G‐A)) were found in the five types (Figure 2). SNP_316_(C‐T) is a C/T mutation located at 316 bp downstream of the ATG initiation site, which was found in the *japonica* group (Figure 2). SNP_316_(C‐T) showed a significant association with HD, SBN, GNPP, TGW and GWSP in the 2016 LD experiment (Table 4). SNP_495_(C‐G) and SNP_614_(G‐A) are major SNPs in the *indica* group. SNP_495_(C‐G) only showed a significant association with HD in the 2016 LD experiment, while SNP_614_(G‐A) exhibited a significant association with SBN, GNPP and GWSP in the 2016 LD experiment and with TGW in the LD and SD conditions (Table 4). SNP_495_(C‐G) is the only difference between haplotypes H14 and H15, but there was no significant difference among the eight yield‐related traits between these haplotypes. However, a significant difference was found between haplotype H16 (one base substituted and four base (AAAG) deficiency with H14, four base (AAAG) deficiency with H15) and H14 or H15 among the eight yield‐related traits (Table S3).

**Table 4:** *Hd1* haplotype associations with eight agronomic traits

Site	Year	C316T		C495G		G614A	
Traits		*P*	*R* ^2^	*P*	*R* ^2^	*P*	*R* ^2^
HD	2016 LD	0.0495	0.2149	0.0154	0.3543	n.s.	–
	2017 SD	n.s.	–	n.s.	–	n.s.	–
TN	2016 LD	n.s.	–	n.s.	–	n.s.	–
	2017 SD	n.s.	–	n.s.	–	n.s.	–
PBN	2016 LD	n.s.	–	n.s.	–	n.s.	–
	2017 SD	n.s.	–	n.s.	–	n.s.	–
SBN	2016 LD	0.0275	0.2115	n.s.	–	0.0403	0.1868
	2017 SD	n.s.	–	n.s.	–	n.s.	–
GNPP	2016 LD	0.0146	0.2653	n.s.	–	0.0132	0.2717
	2017 SD	n.s.	–	n.s.	–	n.s.	–
TGW	2016 LD	0.0354	0.2509	n.s.	–	0.0025	0.4458
	2017 SD	n.s.	–	n.s.	–	0.0387	0.2187
GWPP	2016 LD	n.s.	–	n.s.	–	n.s.	–
	2017 SD	n.s.	–	n.s.	–	n.s.	–
GWSP	2016 LD	0.0263	0.1733	n.s.	–	0.0263	0.1733
	2017 SD	n.s.	–	n.s.	–	n.s.	–

Result of structure‐based association mapping (*P *<* *0.05) of haplotypes 8 and 13–16 by GLM analysis of TASSEL. *R* ^2^, the total variation explained by the SNP.

### Improvement of *Hd1* alleles in *indica*‐*japonica* breeding

**Figure 5:**
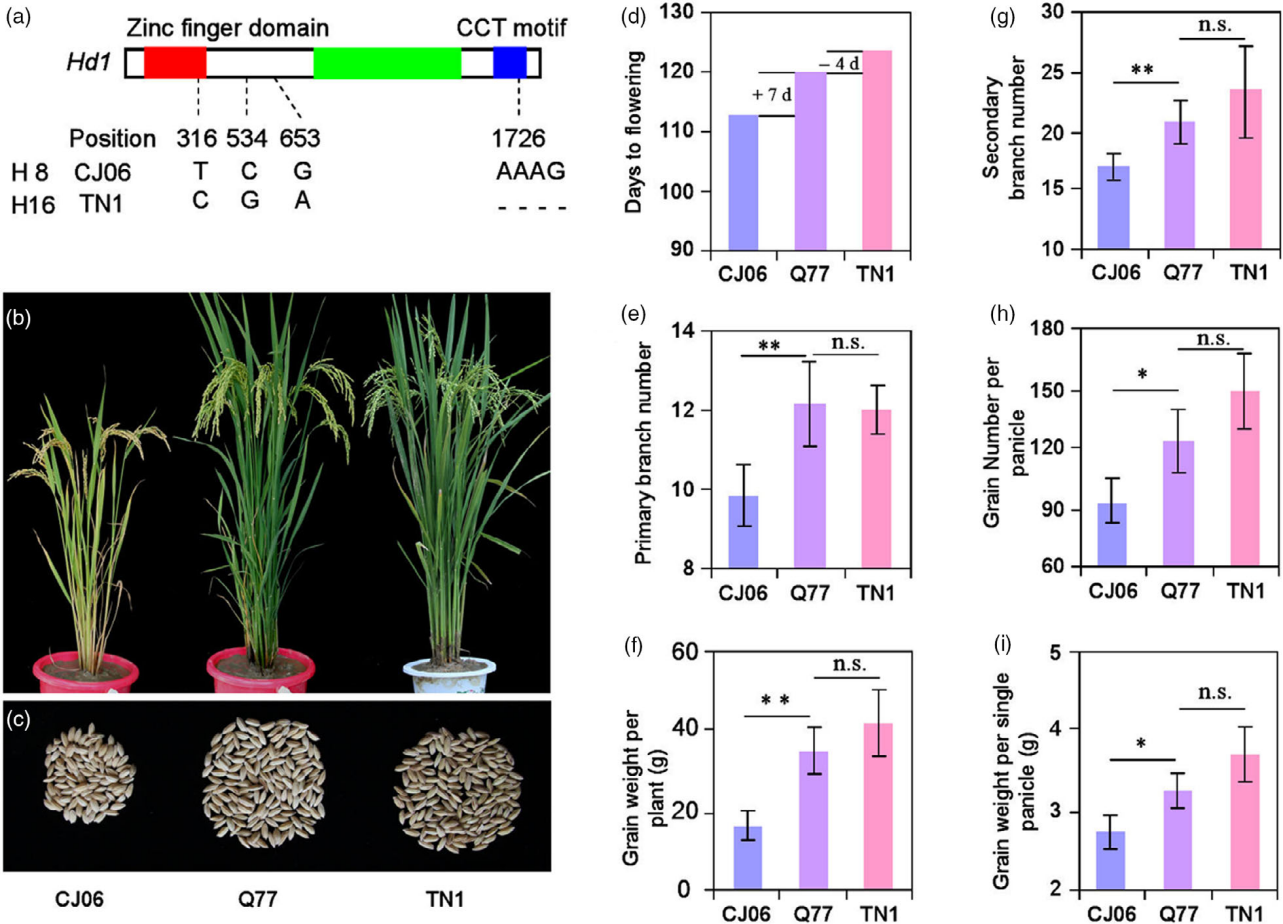
Improvement of *Hd1* alleles between *japonica* and *indica*. (a) Sequence alignment in *Hd1* between CJ06 and TN1. The zinc finger domain, CCT motif and intron are indicated by red, blue and green rectangles, respectively. The number and letter indicate the base position and difference, respectively. (b) Phenotypes of CJ06, Q77 and TN1 plants at the heading stage in the SD condition. (c) Phenotypes of grain weight per plant in CJ06, Q77 and TN1 plants. The comparison of heading date (d), primary branch number (e), grain weight per plant (f), secondary branch number (g), grain number per panicle (h) and grain weight per single panicle (i) in CJ06, Q77 and TN1 plants. Error bars indicate SD; *P <* *0.05, **P <* *0.01 (Student's *t* test).

Due to the influence of photosensitivity, the heading date of *japonica* cultivars was gradually shortened in the process of extending rice cultivation to the south area of China, which resulted in a decrease in grain yield. Therefore, it is necessary to introduce the heading‐related genes of *indica* varieties into *japonica* varieties to prolong the heading date. According to the above results of preponderant *Hd1* alleles of *indica* and *japonica* varieties, we hybridized and screened the progeny of the *japonica* variety Chunjiang06 (CJ06, the same *Hd1* alleles as haplotype H8) and the *indica* variety Taichung native 1 (TN1, the same *Hd1* alleles as haplotype H16; Figure 5a). A BC_4_F_5_ line (Q77) containing the *Hd1* ^*TN1*^ fragment in the CJ06 background was selected (Figure 5b, c). In a comparison of yield‐related traits among CJ06, Q77 and TN1 plants, we found that the HD of Q77 was 7 days longer than that of CJ06 (receptor parent), the number of GNPP, PBN and SBN were significantly increased by 29.50, 2.31 and 3.77, respectively, and the weight of grain weight per plant (GWPP) and GWSP were significantly increased by 18.50 and 0.54, respectively. In contrast, there was no significant difference in these traits between Q77 and TN1 (Figure 5d–i).

**Figure 6:**
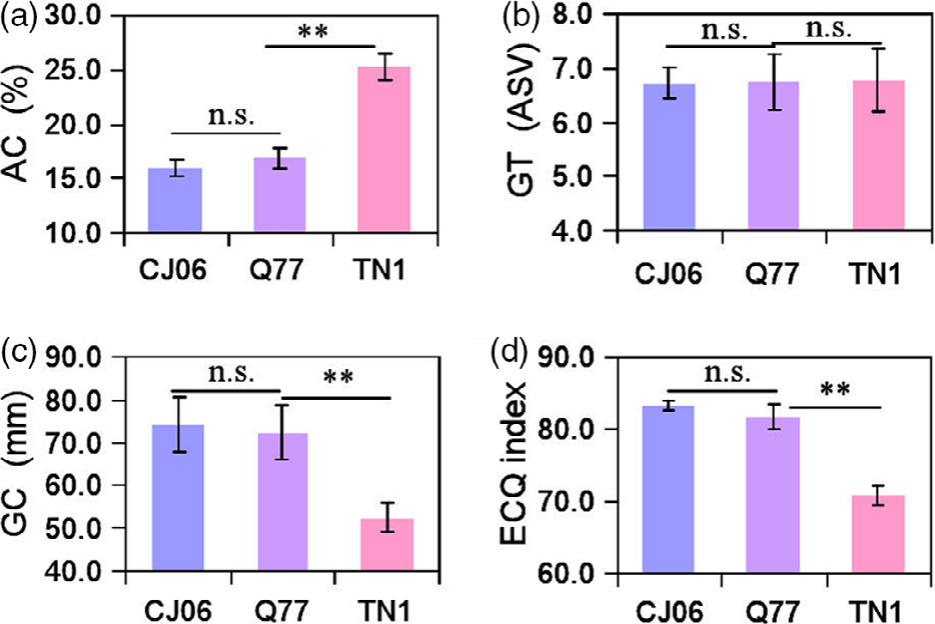
Comparison of eating and cooking quality‐related traits among CJ06, Q77 and TN1. Comparison of amylose content (a), gelatinization temperature (b), gel consistency (c) and ECQs index (d) among CJ06, Q77 and TN1 plants. Error bars indicate SD; **P <* *0.01 (Student's *t* test).

Changing the heading date in rice may affect grain qualities (Cho *et al*., 2013). Therefore, we detected the quality‐related traits (amylose content; AC, gel consistency; GC, gelatinization temperature; GT and eating and cooking qualities; ECQ index) of CJ06 (high quality), Q77 and TN1 (low quality). There were no significant differences between Q77 and CJ06 for AC, GC and the ECQ index, but there was a significant difference between Q77 and TN1 for these traits (Figure 6a, c, d). There were no significant differences among CJO6, Q77 and TN1 for GT (Figure 6b). The results indicated that the introduction of the *Hd1* ^*ind*^ alleles of haplotype H16 into the CJ06 did not change the quality of the grain. Therefore, it is feasible to use the *Hd1* ^*ind*^ alleles of haplotype H16 to prolong the heading date of a *japonica* variety, thereby increasing grain yield without changing quality.

## Discussion

Rice has an estimated 8000‐ to 10 000‐year history of domestication and breeding (Doebley *et al*., 2006; Takahashi *et al*., 2009). Human selection and adaptation to diverse environments have resulted in numerous cultivars (Khush, 1997). Natural genetic variation for heading date has been largely documented, which provides a valuable material for improving adaptation to local environments (Goretti *et al*., 2017). Despite the different alleles of heading date was application in rice traditional breeding programmes, the best gene pyramiding at the molecular level is still unclear. In this study, we selected and sequenced *Hd1* from 123 major rice varieties cultivated from 1936 to 2009 in China to identify the key SNPs in *Hd1* that affect yield‐related traits. Then, we utilized the preponderant alleles of *Hd1* for heading date‐, yield‐ and quality‐related trait improvement.

### The diverse *Hd1* alleles display significant *indica‐japonica* differentiation

*Hd1* is an important gene for the control of flowering time*,* and its sequence has evolved a high degree of polymorphism during rice domestication (Takahashi *et al*., 2009; Wei *et al*., 2014; Zheng *et al*., 2016). Takahashi *et al*. (2009) identified 17 *Hd1* allele types using 64 core rice cultivars worldwide. Zheng *et al*. (2016) reported 39 allele types from 154 rice germplasms. However, these reports did not point out whether *Hd1* has preponderant alleles in *indica*‐*japonica* rice breeding. Our results show that there are obvious differences in the preponderant *Hd1* alleles between *indica* and *japonica* varieties (Figure 4a). Furthermore, our results clearly demonstrated that the preponderant alleles of *Hd1* ^*jap*^ did not change much in the breeding of modern Chinese varieties, but did change notably in *Hd1* ^*ind*^ (Figure 4a, b). In addition, we found that the increase in grain yield in modern *indica* varieties is mostly due to the selection for SBN (Table 3). This result indicates that the rice breeding model in China has focused on the high‐yield model with large panicles, and increasing the number of secondary branches is the key way to generate rice with large panicles.

### Association analysis and utilization of preponderant alleles provide a critical strategy for rice improvement

Candidate gene‐based association mapping takes advantage of recombination events in a natural population to resolve complex trait variation to individual nucleotides (Zhu *et al*., 2008). The selection and utilization of preponderant alleles have become an important way to cultivate high‐yielding varieties. For example, Lu *et al*. (2012) identified a preponderant allele (S_555) of *Ghd7* using 104 rice accessions. Utilization of the alleles (S_555) can decrease plant height and allow the plant to be more resistant to lodging, while not influencing heading date and yield traits in *indica* subspecies. In our study, we successfully used the preponderant alleles of *Hd1* ^*ind*^ haplotype H16 to prolong the heading date of a *japonica* variety CJ06 (Figure 5), indicating that this is a feasible approach to improving the heading date in *japonica* varieties.

*Japonica* varieties are mainly cultivated in the middle and low Yangtze region and northeast China, but this area is only one‐fourth of the total rice planting area of China. The biggest problem with growing *japonica* varieties in lower latitudes is that in lower latitudes they will flower too early and not reach their full potential yield and quality. Therefore, it is an important aim for rice breeding to prolong the heading date of *japonica* varieties appropriately without affecting grain yield and quality. Our results demonstrated that the introduction of the preponderant *Hd1* ^*ind*^ alleles of haplotype H16 into a *japonica* cultivar significantly increased the yield per plant but did not change the quality‐related traits (Figures 5 and 6). This result implied that cultivating *japonica* varieties in the south of China can be achieved by introducing the *Hd1* ^*ind*^ allele of haplotype H16 to improve heading date, yield and quality traits.

## Experimental procedures

### Plant materials and plant growth condition

A total of 123 major rice (*O. sativa* L.) varieties comprising 69 *indica* and 54 *japonica* varieties were selected from the germplasm centres of the China National Rice Research Institute. Most accessions are leading varieties cultivated in China from 1936 to 2009. The basic information for each germplasm appears in Table S1. The experiments were performed at the experimental farm of the China National Rice Research Institute in Hangzhou (HZ, 36°30′N) and Hainan (HN, 18°48′N) during the 2016 and 2017 rice‐growing seasons, respectively. The plants were grown in ten rows with six plants in each row at a planting density of 20 × 20 cm^2^. Field management, including irrigation, fertility and pest control, followed normal agricultural practices.

### Evaluation of traits

Heading date (HD) was defined as the days from sowing to the appearance of the first panicle. Tiller number (TN), primary branch number (PBN), secondary branch number (SBN) and grain number per plant (GNPP) were measured 25 days after heading. GNPP was selected the highest panicle as the grain number. Except for two marginal plants in each side, ten independent plants were used to score the phenotypic data sets. Thirty‐five days after flowering, seeds from each plant were harvested and weighed after physicochemical properties had stabilized. Grain weight was calculated on the basis of 200 grains and converted to TGW. Grain weight per plant (GWPP) was the weight of all seeds harvested from a single plant. Grain weight per single panicle (GWSP) was the weight of the seeds harvested from a single tiller. For quality‐related traits, 15 grains of milled rice were selected for measuring gelatinization temperature (GT), and 15 g of grain was ground to flour to measure amylose content (AC) and gel consistency (GC). AC, GC and GT were measured according to the procedures in Leng *et al*. (2013). The eating and cooking qualities (ECQs) index was measured according to the procedure in Zeng *et al*. (2017).

### DNA extraction, PCR and sequence analysis

Genomic DNA was extracted from fresh leaves of each plant using the cetyltrimethylammonium bromide (CTAB) method (Murray and Thompson, 1980). The polymorphic simple sequence repeat (SSR) markers, randomly distributed across the 12 rice chromosomes, are listed in Table S2. PCRs for amplification of the SSRs were conducted according to the methods in Jin *et al*. (2010) and the products were run on an 8% denaturing polyacrylamide gel at 120 V for 100 min and the gel was visualized using silver staining. *Hd1*, including the 1825‐bp coding region, was amplified from genomic DNA using KOD plus (TOYOBO, Tokyo, Japan). PCRs were conducted using standard PCR protocols. The primers used for PCR and sequencing are listed in Table S4. The initial genomic sequence of *Hd1* was assembled using DNA star software (DNAStar Inc., Madison, WI).

### Genetic diversity and population structure analysis

PowerMarker V3.25 was used to analyse the genetic diversity including the number of alleles per locus, major allele frequency, gene diversity and polymorphism information content (PIC) values (Liu and Muse, 2005). Nei's distance (Nei *et al*., 1983) was calculated and used for the unrooted phylogeny reconstruction using the neighbour‐joining method as implemented in PowerMarker with the tree viewed using MEGA 4.0 (Tamura *et al*., 2007). The population structure among the 123 rice varieties, based on the genotype data, was performed using STRUCTURE V 2.3.4 (Pritchard and Wen, 2004). The number of populations (*K*) was selected from 2 to 10 and five independent runs of a burn‐in of 10 000 iterations followed by 100 000 iterations for each value of *K*. The optimum structure number of *K* was selected based on the report of Evanno *et al*., 2005.

### RNA preparation and qRT‐PCR analysis

Total RNA was extracted using the RNeasy plant mini kit (Qiagen, Valencia, CA) following the manufacturer's instructions. RNA preparations were treated with DNase I (Takara, Tokyo, Japan) to remove traces of DNA contamination. Reverse transcription was conducted with the ReverTra Ace qPCR‐RT Kit (TOYOBO). After synthesis, the cDNA was diluted fivefold in TE buffer, and 1 μL was used for quantitative PCR using the Fast SYBR Green Master Mix (Applied Biosystems, Carlsbad, CA) and gene‐specific primers (Table S4) in an ABI7900 analyser (Applied Biosystems, Foster City, CA).

### The development of chromosome segment substitution lines

The TN1/CJ06 chromosome segment substitution lines (CSSLs) were obtained according to Su *et al*. (2011). The plants carrying the TN1 genotype at the flanking region of *Hd1* were selected to cross with the CJ06 variety for five rounds of backcrossing. A total of 71 SSR markers were used to screen for the genetic background (Ren *et al*., 2016). The SSR markers RM539 and RM454 were used to identify the plants containing the TN1 genotype in backcross progeny.

### Statistical analysis

Analysis of variance was performed using Microsoft Excel 2003. Duncan's multiple comparison was performed by SAS 8.0 software. *Hd1* sequences were aligned by Clustalx 2.1, and the alignment results were input into TASSEL. A general linear model (GLM) was performed in TASSEL V 3.0 for association analysis, which accounted for population structure (Q).

## Author contributions

YJL and YHG performed most of the research. LC, LCH, LPD, DYR, QKX, YZ, KP, LS and GJD performed the trait investigation. YLY, JH, GHZ, GC, ZYG, LBG and GYY analysed the data. YJL wrote the article. YJL, LZ, QQ and DLZ designed the research. DLZ revised the article.

## Competing financial interests

The authors declare that they have no competing financial interests to disclose.

## Supporting information


**Table S1** Basic information for 123 major rice varieties cultivated in China.

**Figure S1** Population structure and unrooted neighbor‐joining trees of 123 major rice varieties cultivated in China.
**Table S2** Summary statistics for the 28 SSR markers used in this study.
**Table S3** Comparison of means of eight traits among the three major *indica* haplotypes.
**Table S4** The primer sequences used in this study.
