# Supplementary material for: Using Heading date 1 preponderant alleles from indica cultivars to breed high‐yield, high‐quality japonica rice varieties for cultivation in south China
Source: Plant Biotechnol J. 2019 Jun 17;18(1):119–28. doi: 10.1111/pbi.13177 (PMC6920332; doi:10.1111/pbi.13177)
Supplement: Supplementary file 2 — Figure S1 Population structure and unrooted neighbor‐joining trees of 123 major rice varieties cultivated in China. Table S2 Summary statistics for the 28 SSR markers used in this study. Table S3 Comparison of means of eight traits among the three major indica haplotypes. Table S4 The primer sequences used in this study. [file PBI-18-119-s002.doc]

**Supporting Information**

**Figure S1** Population structure and unrooted neighbor-joining trees of 123 major rice varieties cultivated in China.

**Table S2** Summary statistics for the 28 SSR markers used in this study.

**Table S3** Comparison of means of eight traits among the three major *indica* haplotypes.

**Table S4** The primer sequences used in this study.


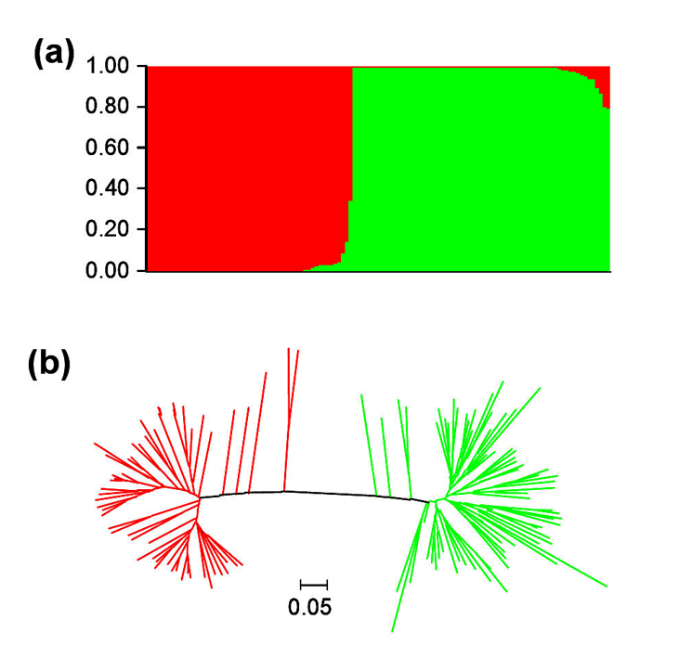


**Figure S1** Population structure and unrooted neighbor-joining trees of 123 major rice varieties cultivated in China.

(a) Population structure of 123 rice varieties. Each variety is represented by a single vertical line with the lengths proportional to each of the subpopulations. The figure is created by STRUCTURE. (b) Unrooted neighbor-joining trees of 123 varieties based on Nei’s genetic distances. Red and green indicate the subpopulations 1 and 2, respectively.

**Table S2** Summary statistics for the 28 SSR markers used in this study.

| Marker | Chr. | Position | Alleles | Gene Diversity | PIC |
| --- | --- | --- | --- | --- | --- |
| RM5302 | 1 | 20.2 | 5 | 0.5837 | 0.499 |
| RM128 | 1 | 134.8 | 6 | 0.6737 | 0.6149 |
| RM1198 | 1 | 146.4 | 9 | 0.6566 | 0.6124 |
| RM211 | 2 | 14.4 | 2 | 0.4997 | 0.3749 |
| RM526 | 2 | 136.3 | 2 | 0.4807 | 0.3651 |
| RM4992 | 3 | 22.1 | 8 | 0.6639 | 0.636 |
| RM1164 | 3 | 68.7 | 7 | 0.6126 | 0.5649 |
| RM411 | 3 | 127.9 | 2 | 0.4682 | 0.3586 |
| RM1352 | 3 | 145.6 | 6 | 0.6682 | 0.6093 |
| RM518 | 4 | 25.5 | 5 | 0.6314 | 0.5579 |
| RM348 | 4 | 160.8 | 2 | 0.4966 | 0.3733 |
| RM3476 | 5 | 101 | 9 | 0.6972 | 0.6583 |
| RM274 | 5 | 126.6 | 2 | 0.496 | 0.373 |
| RM508 | 6 | 2.25 | 3 | 0.5278 | 0.419 |
| RM412 | 6 | 142.4 | 3 | 0.5137 | 0.3982 |
| RM427 | 7 | 11.4 | 3 | 0.5062 | 0.3866 |
| RM172 | 7 | 115.3 | 2 | 0.4992 | 0.3746 |
| RM408 | 8 | 1.1 | 3 | 0.4883 | 0.3773 |
| RM339 | 8 | 72.2 | 5 | 0.6269 | 0.5528 |
| RM444 | 9 | 3.2 | 9 | 0.798 | 0.7735 |
| RM553 | 9 | 76.7 | 2 | 0.4773 | 0.3634 |
| RM1026 | 9 | 93.1 | 5 | 0.6433 | 0.5859 |
| RM216 | 10 | 17.6 | 4 | 0.4489 | 0.4083 |
| RM3470 | 10 | 45.7 | 4 | 0.6797 | 0.6155 |
| RM167 | 11 | 37.5 | 4 | 0.5597 | 0.462 |
| RM224 | 11 | 120.1 | 4 | 0.6483 | 0.5787 |
| RM247 | 12 | 26.7 | 3 | 0.5507 | 0.4643 |
| RM5609 | 12 | 93 | 9 | 0.7407 | 0.7168 |
| Total alleles |  |  | 128 |  |  |
| Total average |  |  | 4.5714 | 0.5835 | 0.5027 |

**Table S3** Comparison of means of eight traits among the three major *indica* haplotypes.

| Trait | Year | H 14 (n=12) | H 15 (n=26) | *P* | H 14 (n=12) | H 16 (n=24) | *P* | H 15 (n=26) | H 16 (n=24) | *P* |
| --- | --- | --- | --- | --- | --- | --- | --- | --- | --- | --- |
|  |  | Means ± SD^a^ | Means ± SD |  | Means ± SD | Means± SD |  | Means ± SD | Means± SD |  |
| HD | 2016 LD | 70.0 ± 6.38 | 74.5 ± 16.93 | 0.3770 | 70.0 ± 6.38 | 85.5 ± 8.41 | < 0.0001^**^ | 74.5 ± 16.93 | 85.5 ± 8.41 | 0.00622^**^ |
|  | 2017 SD | 74.6 ± 1.68 | 75.0 ± 5.89 | 0.7955 | 74.6 ± 1.68 | 91.3 ± 7.22 | < 0.0001^**^ | 75.0 ± 5.89 | 91.3 ± 7.22 | < 0.0001^**^ |
| TN | 2016 LD | 9.0 ± 1.18 | 10.5 ± 2.26 | 0.0363^*^ | 9.0 ± 1.18 | 9.0 ± 1.83b | 0.8802 | 10.5 ± 2.26 | 9.0 ± 1.83 | 0.0185^*^ |
|  | 2017 SD | 6.5 ± 1.09 | 7.3 ± 1.63 | 0.1616 | 6.5 ± 1.09 | 6.1 ± 1.45 | 0.3899 | 7.3 ± 1.63 | 6.1 ± 1.45 | 0.0112^*^ |
| PBN | 2016 LD | 10.1 ± 1.50 | 10.7 ± 2.36 | 0.4424 | 10.1 ± 1.50 | 12.4 ± 1.61 | 0.0002^**^ | 10.7 ± 2.36 | 12.4 ± 1.61 | 0.0038^**^ |
|  | 2017 SD | 9.2 ± 1.03 | 9.9 ± 2.04 | 0.3120 | 9.2 ± 1.03 | 11.5 ± 1.14 | < 0.0001^**^ | 9.9 ± 2.04 | 11.5 ± 1.14 | 0.0009^**^ |
| SBN | 2016 LD | 36.3 ± 9.32 | 30.2 ± 11.44 | 0.1134 | 36.3 ± 9.32 | 42.7 ± 11.66 | 0.1102 | 30.2 ± 11.44 | 42.7 ± 11.66 | 0.0004^**^ |
|  | 2017 SD | 32.5 ± 6.62 | 29.5 ± 11.67 | 0.4247 | 32.5 ± 6.62 | 38.3 ± 10.54 | 0.0885 | 29.5 ± 11.67 | 38.3 ± 10.54 | 0.0076^**^ |
| GNPP | 2016 LD | 178.2 ± 39.31 | 161.2 ± 50.87 | 0.3135 | 178.2 ± 39.31 | 220.5 ± 52.43 | 0.0190^*^ | 161.2 ± 50.87 | 220.5 ± 52.43 | 0.0002^**^ |
|  | 2017 SD | 164.5 ± 32.03 | 163.5 ± 60.68 | 0.9597 | 164.5 ± 32.03 | 206.4 ± 46.19 | 0.0081^**^ | 163.5 ± 60.68 | 206.4 ± 46.19 | 0.0075^**^ |
| TGW | 2016 LD | 25.6 ± 2.64 | 24.5 ± 1.51 | 0.1169 | 25.6 ± 2.64 | 22.6 ± 4.64 | 0.047^*^ | 24.5 ± 1.51 | 22.6 ± 4.64 | 0.0534 |
|  | 2017 SD | 29.0 ± 2.45 | 27.5 ± 2.31 | 0.0810 | 29.0 ± 2.45 | 26.2 ± 5.21 | 0.0926 | 27.5 ± 2.31 | 26.2 ± 5.21 | 0.2597 |
| GWPP | 2016 LD | 40.0 ± 6.90 | 39.6 ± 9.53 | 0.8988 | 40.0 ± 6.90 | 43.1 ± 9.79 | 0.3361 | 39.6 ± 9.53 | 43.1 ± 9.79 | 0.2085 |
|  | 2017 SD | 30.6 ± 5.21 | 30.5 ± 5.84 | 0.9689 | 30.5 ± 5.21 | 31.3 ± 5.12 | 0.6693 | 30.5 ± 5.84 | 31.3 ± 5.12 | 0.5831 |
| GWSP | 2016 LD | 4.6 ± 1.06 | 3.9 ± 1.21 | 0.1388 | 4.5 ± 1.06 | 4.9 ± 1.19 | 0.3918 | 3.9 ± 1.21 | 4.9 ± 1.19 | 0.0064^**^ |
|  | 2017 SD | 4.8 ± 1.02 | 4.4 ± 1.46 | 0.4792 | 4.8 ± 1.02 | 5.3 ± 1.41 | 0.2187 | 4.4 ± 1.46 | 5.3 ± 1.41 | 0.0298^*^ |

H, haplotype; LD, long day; SD, short day; a, SD, standard deviation; N, number of cultivars tested. ^*^ Significantly different at *P*<0.05, ^**^ Significantly different at *P*<0.01 (Student’s *t*-test).

**Table S4** The primer sequences used in this study.

| Primer name | Forward primer | Reverse primer | Remark |
| --- | --- | --- | --- |
| RM5302 | tatgggtgacacattgggac | ttgtgacgtttgagagctgg | Association |
| RM128 | agcttgggtgatttcttggaagcg | acgacgaggagtcgccgtgcag | analysis |
| RM1198 | cttggtcctccacaaagagc | atctgggtcgatcggatatg |  |
| RM211 | ccgatctcatcaaccaactg | cttcacgaggatctcaaagg |  |
| RM526 | cccaagcaatacgtccctag | acctggtcatgacaaggagg |  |
| RM4992 | cagcctgctaatttagtatt | actcgaaatccttctctata |  |
| RM1164 | cgtttctccgagaaaagtcg | caaggtggtcgttgaggc |  |
| RM411 | acaccaactcttgcctgcat | tgaagcaaaaacatggctagg |  |
| RM1352 | acgagttgtactctggttgc | tctcggtttttatcttgctg |  |
| RM518 | ctcttcactcactcaccatgg | atccatctggagcaagcaac |  |
| RM348 | ccgctactaatagcagagag | ggagctttgttcttgcgaac |  |
| RM3476 | gattctcgtcgtaatcaaga | atccacggttaagataaatg |  |
| RM274 | cctcgcttatgagagcttcg | cttctccatcactcccatgg |  |
| RM508 | ggatagatcatgtgtggggg | acccgtgaaccacaaagaac |  |
| RM412 | cacttgagaaagttagtgcagc | cccaaacacacccaaatac |  |
| RM427 | tcactagctctgccctgacc | tgatgagagttggttgcgag |  |
| RM172 | tgcagctgcgccacagccatag | caaccacgacaccgccgtgttg |  |
| RM408 | caacgagctaacttccgtcc | actgctacttgggtagctgacc |  |
| RM339 | gtaatcgatgctgtgggaag | gagtcatgtgatagccgatatg |  |
| RM444 | gctccacctgcttaagcatc | tgaagaccatgttctgcagg |  |
| RM553 | aactccacatgattccaccc | gagaaggtggttgcagaagc |  |
| RM1026 | gcctctggcagaatagcatc | tatcactttgctgcctaggc |  |
| RM216 | gatggtaaaggaagaacgtgtgc | cactcatagacgcatcacatagcc |  |
| RM3470 | tgatgtgatctcctcctggc | agagctgcagaggagacagc |  |
| RM167 | ctccgagtccgaccacaagg | tccagcccttcctatcatattgc |  |
| RM224 | atcgatcgatcttcacgagg | tgctataaaaggcattcggg |  |
| RM247 | tagtgccgatcgatgtaacg | catatggttttgacaaagcg |  |
| RM5609 | cgccagtgtcgaatatgatg | tcttggtgcagtaggtgcac |  |
| Hd-1 | tgcgaggtagaggaacagga | acatacataagaaaaacttccaat | Sequencing |
| Hd-2 | gcaatcaccacacgaaagacc | ctgctatccggaaattacaa |  |
| qHD | tcagcaacagcatatctttctcatca | tctggaatttggcatatctatcacc | qRT-PCR |
| Ubiq | aaccagctgaggcccaaga | acgattgatttaaccagtccatga |  |
